# Photoselective vaporization with green laser versus monopolar transurethral resection for benign prostatic hyperplasia

**DOI:** 10.1590/1806-9282.2024D708

**Published:** 2024-09-02

**Authors:** Antonio Silvinato, Idevaldo Floriano, Wanderley Marques Bernardo

**Affiliations:** 1 Brazilian Medical Association, Evidence-Based Medicine – São Paulo (SP), Brazil.; 2 Universidade de São Paulo, Faculty of Medicine – São Paulo (SP), Brazil.

## DESCRIPTION OF THE EVIDENCE COLLECTION METHOD

The objective was to evaluate the efficacy and safety of photoselective vaporization of the prostate with green light laser (PVP-GL) compared to monopolar transurethral resection (TURP-M) in reducing lower urinary tract symptoms (LUTS) related to benign prostatic hyperplasia (BPH) in a systematic review and meta-analysis of randomized clinical trials (RCTs). The data sources were Medline, CENTRAL/Cochrane, LILACS, and ClinicalTrials.gov (CT.gov) up to February 2024. The eligibility criteria were RCTs comparing the safety and efficacy of PVP-GL versus TURP-M for LUTS and resulting from BPH. The data extracted were perioperative outcomes (surgical time, hospitalization time, and catheterization time); complication rates, including treatment-related adverse events; and functional outcomes, such as International Prostate Symptom Score (IPSS), maximum urinary flow rate (Qmax), and post-void residual volume (PVR). The synthesis was based on the risk differences or pooled mean differences and their corresponding 95% confidence intervals were calculated.

## QUALITY OR CERTAINTY OF EVIDENCE

The certainty of evidence was assessed based on GRADE, graduated in very low, low, moderate, or high.

## GOALS

The objective was to evaluate the efficacy and safety of photoselective vaporization of the prostate with green light laser (PVP-GL) compared to monopolar transurethral resection (TURP-M) in reducing lower urinary tract symptoms (LUTS) related to benign prostatic hyperplasia (BPH) in a systematic review and meta-analysis of randomized clinical trials (RCTs).

## INTRODUCTION

Surgical treatment is one of the cornerstones in managing lower urinary tract symptoms secondary to benign prostatic obstruction. It aims to remove the prostate adenoma through resection, enucleation, or evaporation^1,2^. Transurethral resection of the prostate (TURP), in both monopolar (TURP-M) and bipolar (TURP-B) forms, remains a widely investigated alternative^3^. Due to its widespread availability and effectiveness, TURP-M (the method of choice since the 1970s) is considered the reference technique for the surgical treatment of lower urinary tract symptoms (LUTS)/benign prostatic hyperplasia (BPH) in men with prostates between 30 and 80 mL. The technique removes tissue from the transition zone of the gland in varying degrees, resulting in a reduction in prostate volume and prostate-specific antigen by 25–58%^1,4^. TURP has demonstrated a high success rate and low reintervention rate in long-term follow-up^5^. However, increasing evidence indicates that this invasive procedure is also associated with serious complications such as bleeding, urethral strictures, urinary incontinence, and transurethral resection syndrome (TURS)^6–8^.

In recent years, various techniques have been developed as safe and effective alternatives to TURP-M. One of these is photoselective vaporization of the prostate with PVP-GL. This technique is generally performed with a green laser with a wavelength of 532 nm, generated by potassium-titanyl-phosphate (KTP) or lithium tri borate (LBO) crystals^9^. Unlike other types of lasers, the green laser is easily absorbed by the hemoglobin in soft tissue, while it is hardly incorporated by other fluids (e.g., the irrigant used in the procedure), resulting in better coagulation and a lower risk of injuries to deeper tissues during vaporization^10,11^.

These characteristics also allow the rapid vaporization of prostatic tissue. Photoselective vaporization of the prostate with this laser uses an 80-W KTP generator, a 120-W LBO generator, or a 180-W LBO generator.

This evaluation was conducted to determine whether PVP-GL has advantages over TURP-M in terms of efficacy and safety (perioperative or postoperative outcomes), by rigorously performing a meta-analysis of RCTs. This will provide stronger evidence that will help clinical decision-makers make a more appropriate choice between PVP-GL and TURP-M.

## OBJECTIVE

The objective was to evaluate the efficacy and safety of photoselective vaporization of the prostate with green light laser (PVP-GL) compared to monopolar transurethral resection (TURP-M) in reducing lower urinary tract symptoms (LUTS) related to benign prostatic hyperplasia (BPH). This comparison will be established through a systematic review and meta-analysis of randomized clinical trials (RCTs).

## METHODOLOGY

This assessment is supported by scientific information obtained through a systematic review of the literature, and its conclusions are based on a meta-analysis of the results obtained from the included studies. The exposition of the method used in the systematic review follows the items of the standardized checklist from the Preferred Reporting Items for Systematic Reviews and Meta-Analysis (PRISMA) statement^12^. It has been registered in PROSPERO [PROSPERO (york.ac.uk)], with the registration number CRD42024551534.

### Eligibility criteria

The eligibility criteria define the specific elements to address the clinical question outlined in the objectives of this evaluation, the requirements of greater consistency and scientific strength for study inclusion, and the main reasons for the exclusion of the retrieved evidence.

### Inclusion criteria for studies

- Patients: with lower urinary tract symptoms secondary to benign prostatic hyperplasia, with surgical indication.
- Intervention: selective photovaporization of the prostate with a green light laser.
- Comparison: monopolar transurethral resection of the prostate.
- Outcomes: relevant clinical outcomes of efficacy and safety.
- Study design: double-blind, parallel-controlled RCTs.
- Language: no restrictions.
- Consulted period: no restrictions.
- Full text available.

**Excluded studies:** Crossover RCTs; systematic reviews with or without meta-analysis; narrative reviews; observational studies and/or case series; studies with surrogate endpoints; and the absence of extractable data regarding outcomes (absolute numbers and/or means) or the absence of another study measuring the same outcome, thereby preventing aggregation of their results in the meta-analysis.

### Evidence search

Searches were conducted in the following databases of published scientific information: Medline/PubMed, Cochrane Central Register of Controlled Trials (CENTRAL), LILACS, and ClinicalTrials.gov (CT.gov) for unpublished registry studies. Additional manual searches were performed in the reference lists of included studies and other relevant sources. The search in these databases was conducted till February 2024.

The search strategies used in each database were as follows:

- **Medline/PubMed**—(Prostate OR Prostatic Hyperplasia OR Benign Prostatic Hyperplasia OR Benign Prostate Hyperplasia OR BPH OR Benign Prostatic Hypertrophy OR Prostatic adenoma) AND (Laser Therapy OR Laser Coagulation* OR Laser Thermocoagulation* OR Vaporization OR Volatilization) AND Random*;
- **CENTRAL/Cochrane**—(Prostatic Hyperplasia OR Benign Prostatic Hyperplasia OR BPH) AND (Laser AND Transurethral Resection Prostate);
- **LILACS**—(Prostatic Hyperplasia OR Benign Prostatic Hyperplasia OR BPH) AND (Laser) AND [db: ("LILACS")];
- **ClinicalTrials.gov**—(Prostatic Hyperplasia OR Benign Prostatic Hyperplasia) AND (Laser AND Transurethral Resection Prostate).

### Study selection and data extraction process

The evidence retrieved from the consulted databases is initially selected based on the title and abstract to meet the eligibility criteria. The studies identified in this initial selection then have their full texts accessed to confirm their eligibility. The retrieval process and the evaluation of the obtained titles and abstracts were conducted independently and in a blinded manner by two researchers skilled in systematic reviews (AS and IF), following the inclusion and exclusion criteria. Subsequently, the selected articles were critically evaluated for inclusion in the review. When there was a disagreement about the study selection between the researchers, a third reviewer (WMB) was consulted.

From the eligible studies, the following data will be extracted: the name of the first author and year of publication, the studied population, intervention and comparison methods, and follow-up time. Regarding the extracted data for relevant outcomes, these may include an absolute number of events or means and/or medians with their respective standard deviations or 95% confidence intervals, depending on the type of outcome.

### Risk of bias and quality of evidence

Two independent reviewers assessed the risk of bias in the included studies using the items from the Cochrane Risk of Bias Tool for Randomized Trials (RoB 2)^13^, supplemented by other essential elements, and expressed as high, moderate, and low. Each domain was classified as having no bias, insufficient information, or presence of bias. Publication bias was evaluated through inspection of the funnel plot and by conducting Egger's test^14^.

The Grading of Recommendation, Assessment, Development, and Evaluation (GRADE)^15^ criteria were used as the method to assess the certainty of the effect estimate in the pooled evidence, categorizing the quality of evidence into four levels: high, moderate, low, and very low. Two reviewers evaluated the risk of bias, inconsistency, indirect evidence, imprecision, and publication bias for all reported outcomes. The quality of evidence was assessed using the Guideline Development Tool (GRADEpro GDT)^16^ application and presented in GRADE evidence profiles and summary of findings tables, using standardized terminology.

### Method of analysis and synthesis of results

Data will be analyzed according to the intention-to-treat principle, and the most recent follow-up data available will be included in each trial. The results for categorical outcomes will be expressed using the risk difference (RD) between intervention and control groups, using the Mantel-Haenszel method. If the RD between groups is statistically significant, it will be accompanied by a 95% confidence interval (CI) and the number needed to treat (NNT) or the number needed to harm (NNH). For continuous outcomes, the results will be the mean difference (MD) or standardized mean difference (SMD) if different scales were reported, with a 95%CI.

If there are multiple studies included with common outcomes, they will be pooled using meta-analysis, employing the Review Manager 5.4 (The Nordic Cochrane Centre, The Cochrane Collaboration)^17^. The overall risk difference or mean difference, with 95%CIs, will be the final measure used to support the synthesis of evidence that addresses the clinical question (Objective). For studies that reported data as medians and interquartile ranges, the statistical formula proposed by Hozo et al.^18^ was used to estimate means and standard deviations, in accordance with the methodological guidelines of the Cochrane Handbook for Systematic Reviews^19^. For studies that did not report standard deviation (SD), it will be calculated based on sample size and standard error (SE) or 95%CI.

The estimation of the combined effect size will be conducted using a fixed-effect or random-effects model after assessing the heterogeneity results. Based on statistical heterogeneity findings, the inconsistency was assessed using the I^2^ metric, which measures the percentage of variation attributable to the difference among studies rather than random variation^20^. Heterogeneity values greater than 50% were considered high. A sensitivity analysis was performed to assess the reliability of the findings of this study. A funnel plot was used to analyze asymmetry, which was evaluated after excluding outliers.

### Evidence synthesis and conclusion

The evidence synthesis will present the results directly from the analyses, considering the benefits, harm, and lack of difference between the use of PVP-GL compared to TURP-M. The conclusions will primarily consider evidence of at least moderate quality, assessing the presence of beneficial or harmful effects. Additionally, it will consider the favorable balance between benefit and harm in patients with lower urinary tract symptoms caused by benign prostatic hyperplasia and surgical indications.

## RESULTS

In seeking evidence, 1,102 articles were retrieved from the Medline, CENTRAL, LILACS, and CT.gov databases. Manual and/or gray literature searches did not identify any additional works. After removing duplicates and selecting based on title and/or abstract, 39 articles met the previously established eligibility criteria (Methodology). The full texts of these 39 articles were accessed for analysis.

After reading the full texts, 13 parallel RCTs with placebo were included to support the conclusions of this assessment^21–33^. Two studies^22,25^ were derived from the same clinical trials but with different follow-up periods. A total of 1,538 patients were involved, with 760 treated with PVP-GL and 778 with TURP-M.

**Figure 1:**
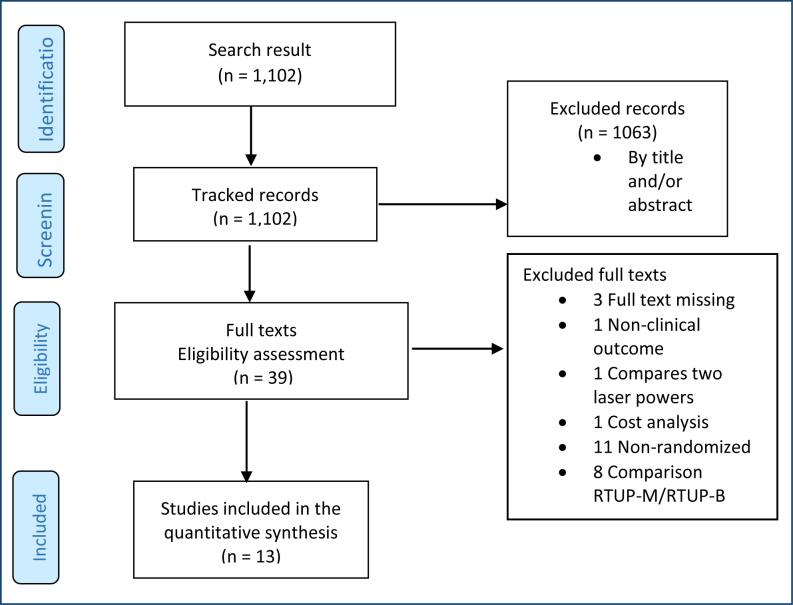
Flow diagram representing the study selection process. From: Moher D, Liberati A, Tetzlaff J, Altman DG, The PRISMA Group. Preferred reporting items for systematic reviews and meta-analyses: the PRISMA statement. PLoS Med. 2009;6(7):e1000097. https://doi.org/10.1371/journal.pmed1000097

The reasons for excluding the other 26 studies are detailed in Figure 1 and in the References section, under the heading "References of Excluded Studies and Their Reasons." Figure 1 presents a flow diagram illustrating the sequence from the retrieval to the selection of evidence for this assessment. The main baseline characteristics and details of each included trial are reported in Table 1 (Appendices).

### Risk of bias in the studies

Of the 13 RCTs included^21–33^, only one study reported blinding of the assessors but did not perform a sample size calculation^27^ (with 10 patients); four studies did not conduct an intention-to-treat (ITT)^21,22,24,28^, and a total of five studies did not perform a sample size calculation^22,24,26,27,33^. The risk of bias assessment for each individual study, using the RoB 2 tool^13^ and additional key elements, is reported in Table 2 (Appendices). The nature of the intervention prevented the blinding of the surgeons. The study was considered double-blinded if patients and outcome assessors were blinded. Any disagreements were resolved by consensus.

## EFFICACY

### Perioperative outcomes

**Figure 2:**
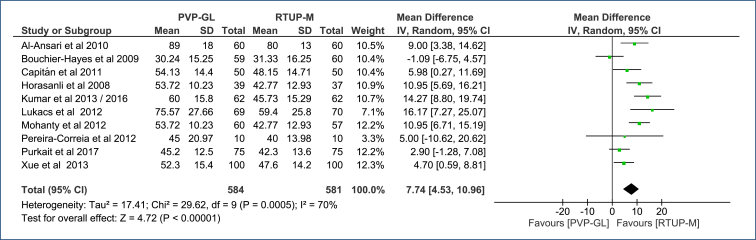
Forest plot of the comparison: 1 Green light laser photoselective vaporization versus monopolar transurethral of the prostate; outcome: 1.1 Surgical time (min).

**Surgical time (min):** Surgical time was recorded in 10 RCTs encompassing a total of 1,165 patients^22,23,26-29,30-33^. There was an average increase of 7.74 min in operation time (MD=7.74 [95%CI, 4.53–10.96]; p<0.00001; I^2^=70%) (Figure 2) with the use of PVP-GL, compared to TURP-M. The certainty of the evidence is moderate (Table 3 in Appendices).

**Figure 3:**
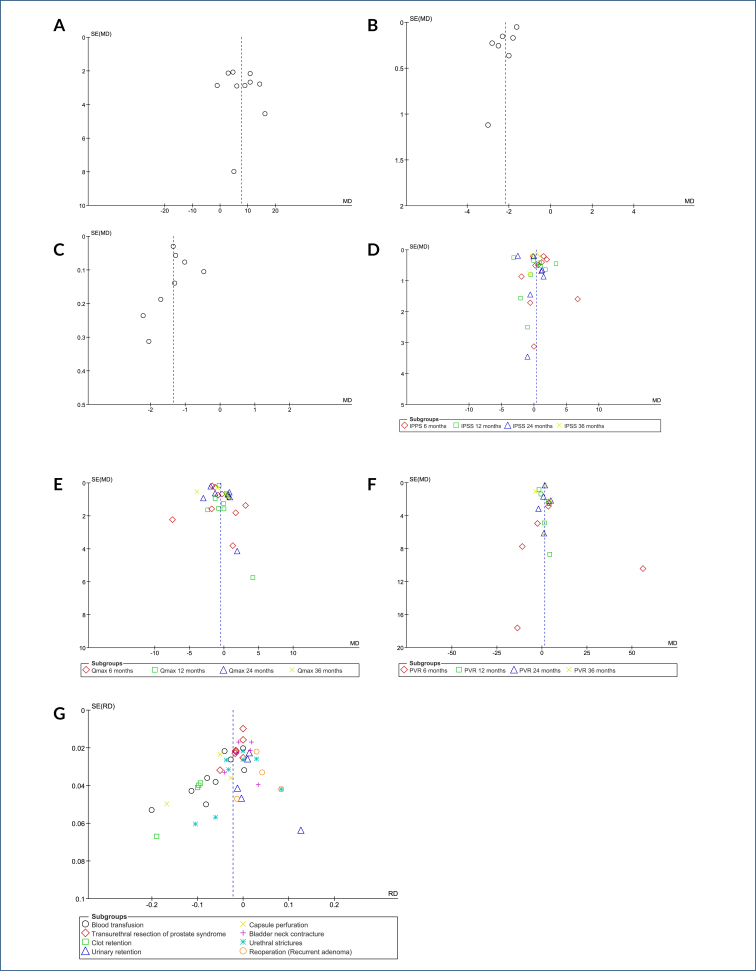
Funnel plots: **(A)** Surgical time. **(B)** Hospitalization time. **(C)** Catheterization time. **(D)** International prostate symptom score. **(E)** Maximum urinary flow rate (Qmax). **(F)** Post-void residual volume (PVR). **(G)** Complications. SE, standard error; MD, mean difference.

The Egger's test (funnel plot) did not identify any outlier studies that would justify the observed heterogeneity (publication bias) (Figure 3 in Appendices). The 70% heterogeneity (I^2^) was not altered with sensitivity analysis due to the absence of outlier studies and/or publication bias.

**Figure 4:**
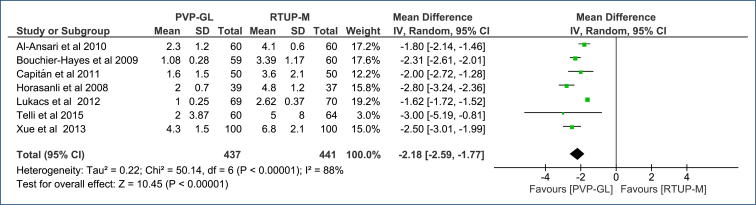
Forest plot of the comparison: 1 Green light laser photoselective vaporization versus monopolar transurethral of the prostate; outcome: 1.2 Hospitalization time (days).

**Hospitalization time (days):** Hospitalization time was reported in seven RCTs encompassing a total of 878 patients^24,26,29,30-33^. PVP-GL, compared to TURP-M, reduces hospitalization time by an average of 2 days (MD=-2.18 [95%CI, −2.59 to −1.77]; p<0.0001; I^2^=88%) (Figure 4). The certainty of the evidence is low (Table 3 in Appendices).

The Egger's test did not identify any outlier studies that would justify the observed heterogeneity (Figure 3 in Appendices). The high heterogeneity (I^2^=88%) was not altered with sensitivity analysis due to the absence of outlier studies and/or publication bias.

**Figure 5:**
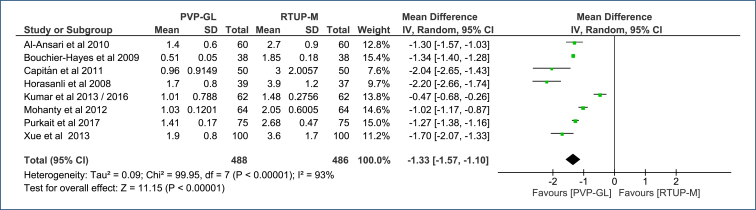
Forest plot of the comparison: 1 Green light laser photoselective vaporization versus monopolar transurethral of the prostate; outcome: 1.3 Catheterization time (days).

**Catheterization time (days):** Catheterization time was reported in eight RCTs, encompassing a total of 974 patients^22,23,26,28,30-33^. Compared to TURP-M, PVP-GL reduces catheterization time by an average of 1 day (MD=-1.33 [95%CI, −1.57 to −1.10]; p<0.0001; I^2^=93%) (Figure 5). The certainty of the evidence is low (Table 3 in Appendices).

The Egger's test did not identify any outlier studies that would justify the observed heterogeneity (Figure 3 in Appendices). The extreme heterogeneity across this sample (I^2^=93%) was not altered with sensitivity analysis due to the absence of outlier studies and/or publication bias.

### Functional outcomes

Initial data, including IPSS, Qmax, and PVR for all participants in the PVP-GL and TURP-M groups, were similar (Table 1 in Appendices).

**Prostate symptoms:** In a subgroup analysis by follow-up time (6, 12, 24, and 36 months), prostate symptoms were evaluated using the IPSS, with a total score ranging from 0 to 35, classifying patients from asymptomatic to very symptomatic.

**Figure 6:**
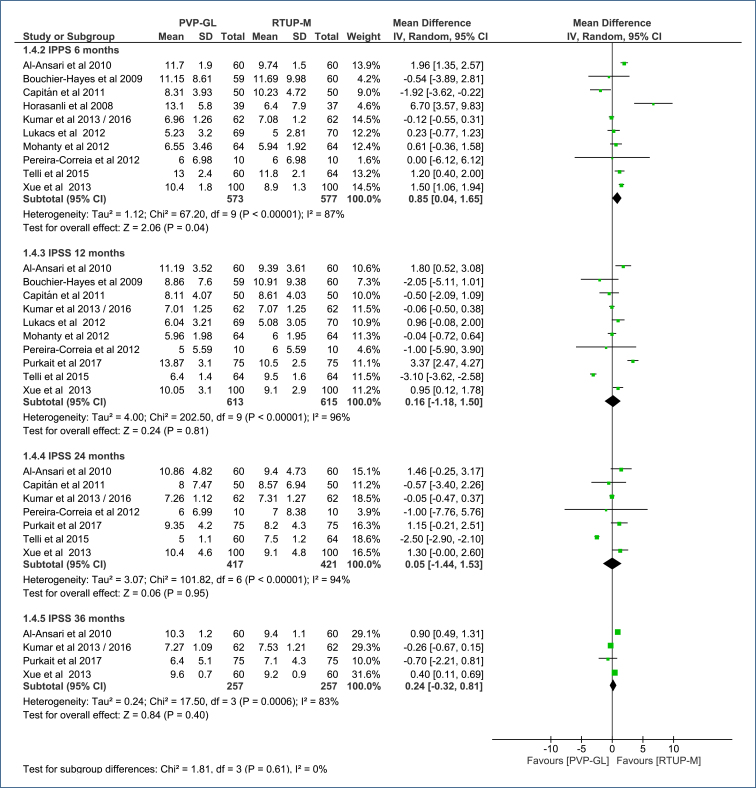
Forest plot of the comparison: 1 Green light laser photoselective vaporization versus monopolar transurethral of the prostate; outcome: 1.4 International Prostate Symptom Score.

At 6 months, compared to TURP-M, PVP-GL showed a less favorable effect, resulting in an average increase of 0.85 points in the IPSS score (MD=0.85 [95%CI, 0.04–1.65]; p=0.04; I^2^=87%) (Figure 6). The certainty of evidence for this difference was classified as low (Table 4 in Appendices).

At 12, 24, and 36 months, there was no difference in the IPSS between the two procedures (p>0.05 for all comparisons) (Figure 6). The certainty of evidence for this lack of difference is very low (Table 4 in Appendices).

The Egger's test did not identify any outlier studies that would justify the observed heterogeneity (Figure 3 in Appendices). High heterogeneity was observed across all follow-up periods (87–94%), but this was not altered by sensitivity analysis due to the absence of outlier studies and/or publication bias.

**Maximum urinary flow rate (Qmax, mL/s):** In 1998, the International Continence Society (ICS) defined Qmax values above 15 mL/s as normal, values between 10 and 15 mL/s as inconsistent, and values below 10 mL/s as pathological^34^.

**Figure 7:**
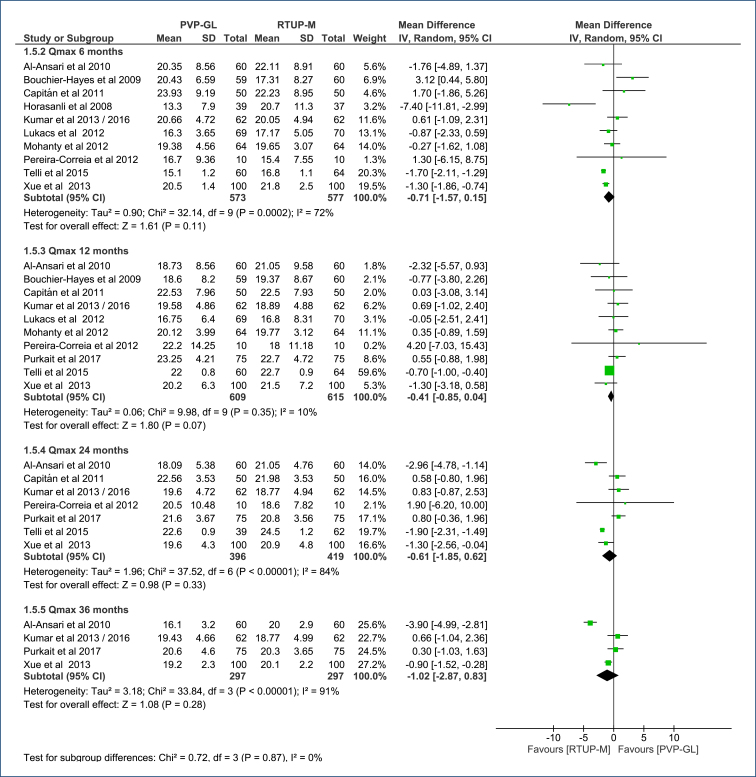
Forest plot of the comparison: 1 Green light laser photoselective vaporization versus monopolar transurethral of the prostate; outcome: 1.5 Qmax (mL/s).

A subgroup analysis by follow-up time (6, 12, 24, and 36 months) evaluated Qmax. At no time points during follow-up, there was a difference in Qmax between the two procedures (p>0.05 for all comparisons) (Figure 7). The certainty of evidence for this lack of difference ranged from low to very low (Table 4 in Appendices).

The Egger's test did not identify any outlier studies that would justify the observed heterogeneity (Figure 3 in Appendices). There was high heterogeneity in the 6-, 24-, and 36-month follow-ups (72–91%), but this heterogeneity was not altered by sensitivity analysis due to the absence of outlier studies and/or publication bias.

**Post-void residual volume (PVR, mL):** A subgroup analysis by follow-up time (6, 12, 24, and 36 months) including six, six, five, and four RCTs, respectively, assessed PVR.

**Figure 8:**
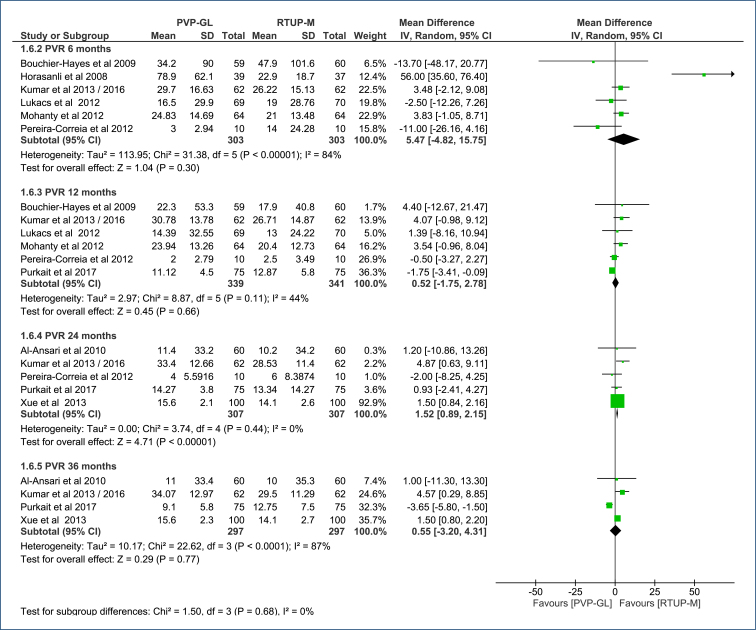
Forest plot of the comparison: 1 Green light laser photoselective vaporization versus monopolar transurethral resection of the prostate; outcome: 1.6 PVR (mL).

At 6 months, there was no difference between the two groups (MD=5.47 mL [95%CI, −4.82 to 15.75 mL]; p=0.30; I^2^=84%). At 12 months, there was no difference either (MD=0.52 mL [95%CI, −1.75 to 2.78 mL]; p=0.66; I^2^=44%). At 36 months, there was no difference in PVR (MD=0.55 mL [95%CI, −3.20 to 4.31 mL]; p=0.77; I^2^=87%) (Figure 8). The evidence certainty ranged from low to very low (Table 4 in Appendices).

At 24 months, PVP-GL has a less favorable outcome, increasing the PVR by 1.52 mL (MD=1.52 [95%CI, 0.89–2.5 mL]; p=0.00001; I^2^=0%) (Figure 8). The evidence certainty was moderate (Table 4 in Appendices).

The Egger's test identified studies with divergent results that justified the observed heterogeneity at 6 and 36 months (Figure 3 in Appendices). To evaluate the influence of these studies, a sensitivity analysis was performed.

At 6 months, the study by Horasanli et al. was removed due to a much larger effect compared to other studies. This adjustment decreased heterogeneity (I^2^=24%) but did not change the significance of the difference in PVR between the procedures.

At 36 months, the study by Purkait et al. was removed due to a result contradicting the other studies. This adjustment eliminated the heterogeneity (I^2^=0%) and increased the MD to 1.58 mL (95%CI, 0.89–2.26 mL; p<0.00001). This result, like the 24-month observation, was unfavorable to PVP-GL.

## SAFETY

### Perioperative and late complications

**Figure 9:**
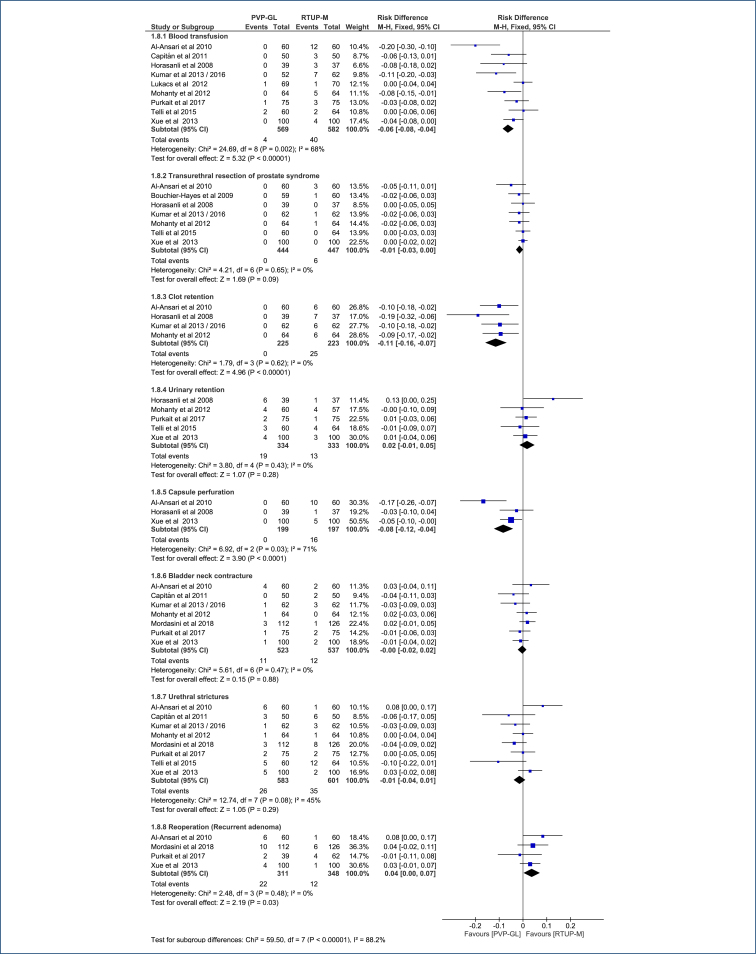
Forest plot of the comparison: 1 Green light laser photoselective vaporization versus monopolar transurethral resection of the prostate; outcome: 1.7 Complications.

In comparison with TURP-M, PVP-GL reduces the risk of blood transfusion by 6.25% (95%CI, 4–8.4%), with 16 patients who need treatment (95%CI, 12–25) to avoid one transfusion (NNT); reduces the risk of clot retention by 11% (95%CI, 7–16%), NNT=9 (95%CI, 7–14); and reduces the risk of capsule perforation by 8% (95%CI, 4–12%), NNT=12 (95%CI, 8–23) (Figure 9). The certainty of the evidence for blood transfusion and clot retention is moderate, while for capsule perforation, it is low (Table 5 in Appendices).

There is no difference between the two procedures for transurethral resection syndrome (DR=0.01 [95%CI, 0.00–0.03]; p=0.09), urinary retention (DR=-0.02 [95%CI, −0.05 to 0.014]; p=0.28), bladder neck contracture (DR=0.001 [95%CI, −0.02 to 0.02]; p=0.88), and urethral stricture (DR=0.01 [95%CI, −0.01 to 0.04]; p=0.29) (Figure 9). The certainty of evidence for urinary retention and bladder neck contracture is moderate, while for transurethral resection syndrome and urethral stricture, it is low (Table 5 in Appendices).

The risk of reoperation for recurrent adenoma was higher with PVP-GL by 4% compared to TURP-M (DR=4% [95%CI, 0.3–7%]; NNH=27 [95%CI, 14–372]; p=0.03; I^2^=0%) (Figure 9). The certainty of evidence is low (Table 5 in Appendices).

Egger's test (funnel plot) identified one study^31^ with discrepant results that accounted for the observed heterogeneity (publication bias) regarding the outcomes of blood transfusion and capsule perforation. Figure 3 (G) in Appendices presents these results. To assess the influence of this study, a sensitivity analysis was conducted.

For the outcome of blood transfusion, the study by Al-Ansai et al. was removed due to its significantly larger effect compared to the others. This adjustment reduced heterogeneity (I^2^ from 68 to 41%) and the risk difference by 1%. The significance of the difference between the procedures remained (DR=5% [95%CI, 0.025–0.07]; p<0.0001; NNT=22 [95%CI, 15–40]), with a still favorable benefit to PVP-GL.

For the outcome of capsule perforation, the study by Al-Ansari et al. was also removed for the same reason as in the blood transfusion outcome. Heterogeneity was reduced from 71 to 0% and the risk difference by 4%. The significance of the difference between the procedures remained (DR=4.4% [95%CI, 0.08–0.10]; p=0.03; NNT=23 [95%CI, 13–104]), as well as the favorable benefit to PVP-GL.

## EVIDENCE SYNTHESIS

### The PVP-GL compared to TURP-M

#### 1. Perioperative outcomes

- Increases the surgical time by an average of 8 min [95%CI, 4.53–10.96]. The certainty of evidence is moderate.
- Reduces the length of hospitalization by an average of 2 days [95%CI, 2.59–1.77]. The certainty of evidence is low.
- Reduces the catheterization time by an average of 1 day [95%CI, 1.57–1.10]. The certainty of evidence is low.

#### 2. Functional outcomes

#### IPSS

- At 6 months, it shows a less favorable effect, as it increases the IPSS score by an average of 0.85 points (95%CI, 0.04–1.65). The certainty of evidence for this difference was classified as low.
- At 12, 24, and 36 months, there is no difference in IPSS (p>0.05 for these comparisons). The certainty of evidence is very low for this lack of difference.

#### Qmax (mL/s)

- There is no difference in Qmax at the 6-, 12-, 24-, and 36-month follow-ups (p>0.05 for these comparisons). The certainty of evidence for this lack of difference varies from low to very low.

#### PVR (mL)

- It does not show a difference at 6, 12, and 36 months (p>0.05 for these comparisons). The certainty of evidence for this lack of difference varies from low to very low.
- At 24 months, it shows a less favorable result, as it increases the PVR by 1.52 mL (95%CI, 0.89–2.5). This response does not persist at 36 months, as seen above. The certainty of evidence for this difference is moderate.

#### 3. Complications (perioperative and late)

- Reduces risk of blood transfusion by 6.25% (95%CI, 4–8.4%), NNT=16 (95%CI, 12–25). The certainty of evidence is moderate.
- Reduces the risk of clot retention by 11% (95%CI, 7–16%), NNT=9 (95%CI, 7–14). The certainty of evidence is moderate.
- Reduces the risk of capsule perforation by 8% (95%CI, 4–12%), NNT=12 (95%CI, 8–23). The certainty of evidence is low.
- Does not show a difference in outcomes related to transurethral resection syndrome of the prostate, urinary retention, bladder neck contracture, and urethral stricture (p>0.05 for these comparisons). The certainty of evidence is moderate for urinary retention and bladder neck contracture, while for transurethral resection syndrome of the prostate and urethral stricture, it is considered low.
- Increases the risk of reoperation for recurrent adenoma by 4% (DR=4% [95%CI, 0.3–7%], NNH=27 [95%CI, 14–372]), and the certainty of evidence is low.

## DISCUSSION

Green light laser photoselective vaporization (PVP-GL) has emerged as a promising technique in the management of benign prostatic hyperplasia, showing favorable results when compared to monopolar transurethral resection of the prostate (TURP-M)^35–37^. Our meta-analysis addressed a variety of perioperative outcomes, functional outcomes, and complications. We provided a comprehensive view of the effectiveness and safety of this technique, including only RCTs using green light lasers (KTP, 532 nm wavelength) for PVP. A separate analysis of the use of 80-W and 120-W lasers was challenging due to the scarcity of available data. Therefore, despite well-known limitations and subsequent improvements in the laser, these were considered similar interventions for the purposes of this meta-analysis.

Regarding perioperative outcomes, we observed that PVP-GL increases the average procedure time by 8 min. Although this increase is statistically significant (MD=7.74 min [95%CI, 4.53–10.96 min]; p<0.00001), it is important to note that the difference is moderate and may not be clinically relevant. Additionally, the average reduction of 2 days in hospitalization time and 1 day in catheterization time, although statistically significant, are based on low-certainty evidence, which requires caution in interpreting these results.

PVP-GL showed mixed results compared to TURP-M for functional outcomes. We observed that PVP-GL showed an average increase in IPSS score at 6 months (MD=0.85 [95%CI, 0.04–1.65]; p=0.04), but this difference did not persist in subsequent follow-ups at 12, 24, and 36 months. The lack of significant difference in IPSS in the long term suggests that PVP-GL maintains comparable results to TURP-M over time.

Similarly, there were no significant differences in Qmax and PVR at different follow-ups, highlighting the equivalence of these techniques in terms of functional performance. Sensitivity analysis for IPSS and Qmax outcomes did not identify outlier studies and/or publication bias, maintaining high heterogeneity at follow-up periods. However, for RVR outcome, discrepant studies were identified at 6 and 36 months. Removing these studies resulted in changes in heterogeneity, but not with the same significance as the result at 6 months; at 36 months, the elimination of heterogeneity was accompanied by a less favorable result for PVP-GL (increased MD to 1.58 mL [95%CI, 0.89–2.26 mL; p<0.00001]), although it is a small difference and may not be clinically relevant.

Regarding complications, PVP-GL showed significant advantages. Reductions in the risk of blood transfusion (DR=6.25% [95%CI, 4–8.4%], NNT=16), clot retention (DR=11% [95%CI, 7–16%], NNT=9), and capsule perforation (DR=8% [95%CI, 4–12%], NNT=12) were observed, with moderate certainty evidence. However, no significant differences were found in other complications such as transurethral resection syndrome, urinary retention, bladder neck contracture, and urethral stricture, although the certainty of evidence ranges from moderate to low. PVP-GL increases the risk of reoperation for recurrent adenoma by 4% (DR=4% [95%CI, 0.3–7%], NNH=27 [95%CI, 14–372]).

In summary, our analysis suggests that PVP-GL offers advantages in terms of recovery time and perioperative complications, with comparable functional outcomes to TURP-M in the long term. However, it is important to recognize the limitations of the available evidence, especially regarding perioperative and functional outcomes. For these events, the certainty of evidence is low or very low due to a high risk of bias in the included studies, high heterogeneity, and very wide confidence intervals for many of the outcomes. Despite these limitations, this study provides the most up-to-date information on the comparison of PVP-GL and TURP-M in the surgical treatment of BPH. Future studies with robust designs are needed to confirm and expand these findings, providing a more solid basis, especially in relation to the certainty of evidence, and offering more precise guidelines for clinical practice.

## CONCLUSION

In our meta-analysis of functional outcomes up to 3 years of follow-up after PVP-GL and TURP-M, we found that both procedures showed similar results. Although PVP-GL offers advantages in terms of recovery time and perioperative complications, it is important to highlight the potential risk of reoperation for recurrent adenoma in the long term. However, it is crucial to note that the certainty of evidence available, especially regarding perioperative and functional outcomes, is low or very low.

## APPENDICES

**Table 1:** Key patient baseline characteristics and details of each trial.

First author/year	Patients (N)	Laser power (W)	Age, years	Prostate size (mL)	IPSS	Qmax (mL/s)	PVR (mL)	Follow-up duration (months)
	PVP-GL RTUP-M		PVP-GL (DP) RTUP-M (DP)	PVP-GL (DP) RTUP-M (DP)	PVP-GL (DP) RTUP-M (DP)	PVP-GL (DP) RTUP-M (DP)	PVP-GL (DP) RTUP-M (DP)	
**Al-Ansari31 2010**	60 60	120-w	66.3 ± 9.4 67.1 ± 8.0	61.8 ± 22.0 60.3 ± 20.0	27.2 ± 2.3 27.9 ± 2.7	6.9 ± 2.2 6.4 ± 2.0	53.2 ± 25 57.0 ± 21	1, 3, 6, 12, 24, and 36
**Bouchier-Hayes32 2010**	59 60	80-w	65.1 ± 5.0 66.3 ± 4.2	38.7 ± 11.2 33.4 ± 8.7	25.28 ± 5.93 25.41 ± 5.72	8.81 ± 2.55 8.86 ± 2.99	129.2 ± 155.7 111.3 ± 113.7	3, 6, and 12
**Capitán30 2011**	50 50	120-w	69.8 ± 8.4 67.7 ± 6.7	51.3 ± 14.7 53.1 ± 13.8	23.7 ± 5.2 23.5 ± 4.4	8.0 ± 3.1 3.9 ± 2.7	Não avaliado	1, 3, 6, 12, and 24
**Horasanli33 2008**	39 37	80-w	69.2 ± 7.1 68.3 ± 6.7	86.1 ± 8.8 88.0 ± 9.2	18.9 ± 5.1 20.2 ± 6.8	8.6 ± 5.2 9.2 ± 5.6	183.0±50.1 176.9±45.3	3 and 6
**Kumar22,25 2013/2016**	62 62	120-w	≥ 50 ≥ 50	52.8 ± 16.1 52.2 ± 15.9	20.0 ± 2.7 20.7 ± 2.6	6.68 ± 2.00 7.00 ± 1.97	143.3 ± 52.6 148.4 ± 60.3	1, 3, 6, 12, and 36
**Lukacs29 2012**	69 70	120-w	66.9 ± 7.8 67.6 ± 7.6	50.5 ± 16.5 50.1 ± 14.7	21.7 ± 2.7 19.4 ± 2.4	7.8 ± 2.8 7.8 ± 2.6	89.5 ± 92 75.0 ± 73	1, 3, 6, and 12
**Mohanty28 2012**	64 64	80-w	66.9 ± 8.62 65.7 ± 9.09	44.7 ± 14.09 49.0 ± 15.93	19.9 ± 3.27 20.8 ± 3.87	7.4 ± 2.07 6.7 ± 1.63	145.8 ± 70.33 143.2 ± 65.96	1, 3, 6, and 12
**Mordasini21 2018**	112 126	80-w	68.4 ± 8.7 67.6 ± 8.4	36.1 ± 11.5 37.9 ± 14.3	20.3 ± 7.0 20.4 ± 7.5	8.9 ± 4.1 8.5 ± 4.6	91.1 ± 88.3 114.5 ± 36.4	60
**Pereira27 2012**	10 10	120-w	64.0 ± 6.0 67.0 ± 5.5	46.4 ± 7.1 45.6 ± 7.2	21.1 ± 3.1 20.6 ± 2.8	8.4 ± 3.4 7.9 ± 2.8	109.8 ± 103.9 116.6 ± 78.5	1, 3, 6, 9, 12, and 24
**Purkait23 2017**	75 75	120-w	63.6 ± 8.12 65.3 ± 7.86	70.3 ± 15.5 69.6 ± 16.3	26.1 ± 4.8 25.9 ± 5.2	8.5 ± 2.7 8.3 ± 2.4	238.0 ± 31.0 213.0 ± 23.0	12, 24, 36, and 48
**Teli24 2015**	60 64	120-w	67.0 ± 9.1 69.0 ± 7.8	60.7 ± 8.1 55.7 ± 8.1	20.0 ± 2.7 19 ± 2.6	10.6 ± 3.0 12.5 ± 4.5	60.5 ± 104.1 65.2 ± 100.5	6, 12, and 24
**Xue26 2013**	100 100	120-w	72.1 ± 11.3 71.0 ± 10.8	65.8 ± 23.6 67.3 ± 24.7	23.0 ± 5.1 23.2 ± 5.0	8.0 ± 3.6 8.2 ± 3.8	Não avaliado	1, 3, 6, 12, 24, and 36

Continuous variables were expressed as (mean±SD). PVP-GL, photoselective vaporization of the prostate with green-light laser; RTUP-M, monopolar transurethral resection of the prostate; IPSS, International Prostate Symptom Score; Qmax, maximum flow rate; PVR, post-void residual volume.

**Table 2:** Risk of bias in studies.

First author/Year (Ref. #)	Randomization	Blind allocation	Double-blind	Outcome researcher blind	Losses	Prognostic characteristics	Appropriate outcomes	Intention to treat analysis	Sample size calculation	Early interruption	Global risk of viruses
Al-Ansari A^31^, 2010											HIGH
Bouchier-Hayes DM^32^, 2010											HIGH
Capitán C^30^, 2011											HIGH
Horasanli K^33^, 2008											HIGH
Kumar A^22,25^, 2013/2016											HIGH
Lukacs B^29^, 2012											HIGH
Mohanty NK^28^, 2012											HIGH
Mordasini L^21^, 2018											HIGH
Pereira-Correia JA^27^, 2012											HIGH
Purkait B^23^, 2017											HIGH
Telli O^24^, 2015											HIGH
Xue B^26^, 2013											HIGH
LEGENDA	LOW RISK			NOT INFORMED				HIGH RISK			

**Table 3:** GRADE: Perioperative outcomes.

Summary of findings: Perioperative outcomes			
**Patient or population:** Benign prostatic hyperplasia			
**Setting:** Therapeutic efficacy, safety			
**Intervention:** Photoselective vaporization of the prostate with green light laser (PVP-GL)			
**Comparison:** Transurethral resection of the prostate in the monopolar form (TURP-M)			
**Outcomes**	**Anticipated absolute effects** * **(95% CI)** **Mean difference**	**No. of participants (studies)**	**Certainty of the evidence (GRADE)**
Operation time (min)	MD **7.74 higher** (4.53 higher to 10.96 higher)	1165 (10 RCTs)	Moderate^a^
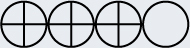
Hospitalization time (days)	MD **2.18 lower** (2.59 lower to 1.77 lower)	878 (7 RCTs)	Low^b^,^c^
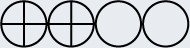
Catheterization time (days)	MD **1.33 lower** (1.57 lower to 1.1 lower)	974 (8 RCTs)	Low b,d
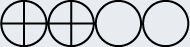

* **The risk in the intervention group** (and its 95% confidence interval) is based on the assumed risk in the comparison group and the **relative effect** of the intervention (and its 95% CI). CI, confidence interval; MD, mean difference.

a There was no blinding of the patient in any study and only one blinded the evaluator. Two studies had >20% loss.

b There was no blinding of the patient and the evaluator in any study and one had a loss greater than 20%.

c I^2^ = 88% and sensitivity analysis does not justify heterogeneity.

d I^2^ = 93% and sensitivity analysis does not justify heterogeneity. **GRADE Working Group grades of evidence** **High certainty:** We are very confident that the true effect lies close to that of the estimate of the effect. **Moderate certainty:** We are moderately confident in the effect estimate: The true effect is likely to be close to the estimate of the effect, but there is a possibility that it is substantially different. **Low certainty:** Our confidence in the effect estimate is limited: The true effect may be substantially different from the estimate of the effect. **Very low certainty:** We have very little confidence in the effect estimate: The true effect is likely to be substantially different from the estimate of effect.

**Table 4:** GRADE: Functional outcomes.

Summary of findings: functional outcomes			
**Patient or population:** Benign prostatic hyperplasia			
**Setting:** Therapeutic efficacy, safety			
**Intervention:** Photoselective vaporization of the prostate with green light laser (PVP-GL)			
**Comparison:** Transurethral resection of the prostate in the monopolar form (TURP-M)			
**Outcomes**	**Anticipated absolute effects** * **(95% CI) Mean difference**	**No. of participants (studies)**	**Certainty of the evidence (GRADE)**
IPSS - IPPS 6 months	MD **0.85 higher** (0.04 higher to 1.65 higher)	1150 (10 RCTs)	Low^b^,^c^
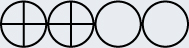
IPSS - IPSS 12 months	MD **0.16 higher** (1.18 lower to 1.5 higher)	1228 (10 RCTs)	Very Low^a^,^d^,^e^
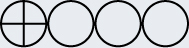
IPSS - IPSS 24 months	MD **0.05 higher** (1.44 lower to 1.53 higher)	838 (7 RCTs)	Very Low^e^,^f^,^g^
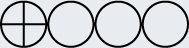
IPSS - IPSS 36 months	MD **0.24 higher** (0.32 lower to 0.81 higher)	514 (4 RCTs)	Very Low^b^,^e^,^h^
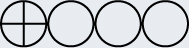
Qmax (mL/s) - Qmax 6 months	MD **0.71 lower** (1.57 lower to 0.15 higher)	1150 (10 RCTs)	Low b,e
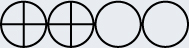
Qmax (mL/s) - Qmax 12 months	MD **0.41 lower** (0.85 lower to 0.04 higher)	1224 (10 RCTs)	Low b,e
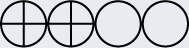
Qmax (mL/s) - Qmax 24 months	MD **0.61 lower** (1.85 lower to 0.62 higher)	815 (7 RCTs)	Very Low^b^,^e^,^i^
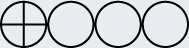
Qmax (mL/s) - Qmax 36 months	MD **1.02 lower** (2.87 lower to 0.83 higher)	594 (4 RCTs)	Very Low b,e,j
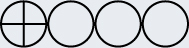
PVR (mL) - PVR 6 months	MD **5.47 higher** (4.82 lower to 15.75 higher)	606 (6 RCTs)	Very Low^e^,^f^,^k^
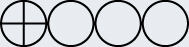
PVR (mL) - PVR 12 months	MD **0.52 higher** (1.75 lower to 2.78 higher)	680 (6 RCTs)	Low e,f
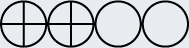
PVR (mL) - PVR 24 months	MD **1.52 higher** (0.89 higher to 2.15 higher)	614 (5 RCTs)	Moderate^f^
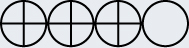
PVR (mL) - PVR 36 months	MD **0.55 higher** (3.2 lower to 4.31 higher)	594 (4 RCTs)	Very Low^b^,^c^,^l^
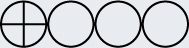

* **The risk in the intervention group** (and its 95% confidence interval) is based on the assumed risk in the comparison group and the **relative effect** of the intervention (and its 95% CI). CI, confidence interval; MD, mean difference.

a There was no blinding of the patient in any study and only one blinded the evaluator. Two studies had >20% loss.

b There was no blinding of the patient and the evaluator in any study and one had a loss >20%.

c I^2^ = 87%, and sensitivity analysis does not justify heterogeneity.

d I^2^ = 96%, and sensitivity analysis does not justify heterogeneity.

e Wide confidence interval.

f There was no blinding of the patients and only one study blinded the evaluator. One study with a loss of more than 20%.

g I^2^ = 94%, but the sensitivity analysis justifies the heterogeneity.

h I^2^ = 83%, and sensitivity analysis does not justify heterogeneity.

i I^2^ = 84%, and sensitivity analysis does not justify heterogeneity.

j I^2^ = 91%, but the sensitivity analysis justifies the heterogeneity.

k I^2^ = 84%, but the sensitivity analysis justifies the heterogeneity.

l Very wide confidence interval.

**Table 5:** GRADE: complications outcomes.

Summary of findings: complications outcomes					
**Patient or population:** Benign prostatic hyperplasia					
**Setting:** Therapeutic efficacy, safety					
**Intervention:** Photoselective vaporization of the prostate with green light laser (PVP-GL)					
**Comparison:** Transurethral resection of the prostate in the monopolar form (TURP-M)					
**Outcomes**	**Anticipated absolute effects** * **(95% CI)**		**Risk difference (95% CI)**	**No. of participants (studies)**	**Certainty of the evidence (GRADE)**
	**With PVP-GL**	**With TURP-M**			
Blood transfusion	4/569 (0.7%)	**40/582 (6.9%)**	-0.06 [-0.08, −0.04]	1151 (9 RCTs)	Moderate^b^
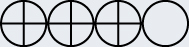
Transurethral resection of prostate syndrome	0/444 (0%)	**6/447 (1.3%)**	-0.01 [-0.03, 0.00]	891 (7 RCTs)	Low^b^,^c^
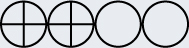
Clot retention	0/225 (0%)	**25/223 (2%)**	-110/1000 [-160, −70]	448 (4 RCTs)	Moderate^d^
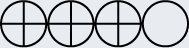
Urinary retention	19/334 (5.7%)	**13/333 (3.9%)**	-0.11 [-0.16, −0.07]	667 (5 RCTs)	Moderate^b^
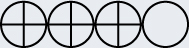
Capsule perforation	0/199 (0%)	**16/197 (8.1%)**	-0.08 [-0.12, −0.04]	396 (3 RCTs)	Low^d^,^e^
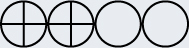
Bladder neck contracture	11/523 (2.1%)	**12/537 (2.2%)**	-0.00 [-0.02, 0.02]	1060 (7 RCTs)	Moderate^a^
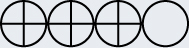
Urethral strictures	26/583 (4.5%)	**35/601 (5.8%)**	-0.01 [-0.04, 0.01]	1184 (8 RCTs)	Low^c^,^f^
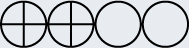
Reoperation (recurrent adenoma)	22/311 (4.5%)	**12/348 (3.4%)**	0.04 [0.00, 0.07]	659 (4 RCTs)	Low^c^,^g^
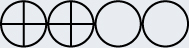

* **The risk in the intervention group** (and its 95% confidence interval) is based on the assumed risk in the comparison group and the **relative effect** of the intervention (and its 95% CI). CI, confidence interval.

a There was no blinding of the patient in any study and only one study blinded the evaluator. Two studies had >20% loss.

b There was no blinding of the patient and the evaluator in any study and one had >20% loss.

c Wide confidence interval.

d There was no blinding of the patient and the evaluator in any study.

e I^2^=71%, but the sensitivity analysis justifies the heterogeneity.

f There was no blinding of the patients and only one study blinded the evaluator. Two studies had >20% loss.

g There was no blinding of the patients and the evaluator. Two studies had >20% loss.
